# *N*′-[(*E*)-4-Benz­yloxy-2-hy­droxy­benzyl­idene]-4-nitro­benzohydrazide di­methyl­formamide monosolvate

**DOI:** 10.1107/S1600536813017091

**Published:** 2013-06-26

**Authors:** Bibitha Joseph, M. Sithambaresan, M. R. Prathapachandra Kurup

**Affiliations:** a Department of Applied Chemistry, Cochin University of Science and Technology, Kochi 682 022, India; b Department of Chemistry, Faculty of Science, Eastern University, Sri Lanka, Chenkalady, Sri Lanka

## Abstract

The title compound, C_21_H_17_N_3_O_5_·C_3_H_7_NO, exists in an *E* conformation with respect to the azomethine double bond of the hydrazide mol­ecule. This mol­ecule contains an intra­molecular O—H⋯N hydrogen bond, while an inter­molecular N—H⋯O hydrogen bond links the hydrazide to the formamide mol­ecule of solvation. Nonclassical C—H⋯O inter­molecular hydrogen bonds build up a supra­molecular architecture, together with two C—H⋯π inter­actions and a weak π–π inter­action, with a centroid–centroid distance of 3.650 (13) Å.

## Related literature

For the biological and analytical applications of carbohydrazides, see: Vicini *et al.* (2002 ▶); Savini *et al.* (2002 ▶); Grande *et al.* (2007 ▶). For the synthesis of related compounds, see: Mathew & Kurup (2011 ▶); Despaigne *et al.* (2009 ▶). For related structures, see: Joseph *et al.* (2012 ▶).
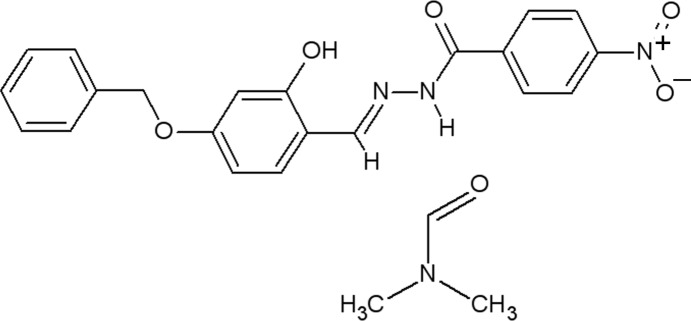


## Experimental

### 

#### Crystal data

C_21_H_17_N_3_O_5_·C_3_H_7_NO
*M* *_r_* = 464.47
Monoclinic, 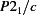

*a* = 10.0160 (8) Å
*b* = 22.661 (2) Å
*c* = 10.2611 (11) Å
β = 101.392 (5)°
*V* = 2283.1 (4) Å^3^
*Z* = 4
Mo *K*α radiation
μ = 0.10 mm^−1^
*T* = 296 K
0.40 × 0.20 × 0.20 mm

#### Data collection

Bruker Kappa APEXII CCD diffractometer
Absorption correction: multi-scan (*SADABS*; Bruker, 2004 ▶) *T*_min_ = 0.977, *T*_max_ = 0.981
16107 measured reflections
4910 independent reflections
2901 reflections with *I* > 2σ(*I*)
*R*_int_ = 0.031

#### Refinement

*R*[*F* ^2^ > 2σ(*F* ^2^)] = 0.053
*wR*(*F* ^2^) = 0.176
*S* = 1.04
4910 reflections
318 parameters
2 restraints
H atoms treated by a mixture of independent and constrained refinement
Δρ_max_ = 0.40 e Å^−3^
Δρ_min_ = −0.22 e Å^−3^

### 

Data collection: *APEX2* (Bruker, 2004 ▶); cell refinement: *APEX2* and *SAINT* (Bruker, 2004 ▶); data reduction: *SAINT* and *XPREP* (Bruker, 2004 ▶); program(s) used to solve structure: *SHELXS97* (Sheldrick, 2008 ▶); program(s) used to refine structure: *SHELXL97* (Sheldrick, 2008 ▶); molecular graphics: *ORTEP-3* (Farrugia, 2012 ▶) and *DIAMOND* (Brandenburg, 2010 ▶); software used to prepare material for publication: *SHELXL97* and *publCIF* (Westrip, 2010 ▶).

## Supplementary Material

Crystal structure: contains datablock(s) I, global. DOI: 10.1107/S1600536813017091/fj2634sup1.cif

Structure factors: contains datablock(s) I. DOI: 10.1107/S1600536813017091/fj2634Isup2.hkl

Supplementary material file. DOI: 10.1107/S1600536813017091/fj2634Isup3.cml

Additional supplementary materials:  crystallographic information; 3D view; checkCIF report

## Figures and Tables

**Table 1:** Hydrogen-bond geometry (Å, °) *Cg*1 is the centroid of the C1–C6 ring.

*D*—H⋯*A*	*D*—H	H⋯*A*	*D*⋯*A*	*D*—H⋯*A*
N2—H2′⋯O6	0.88 (1)	1.95 (1)	2.810 (3)	166 (2)
O2—H2*O*⋯N1	0.85 (1)	1.82 (2)	2.583 (2)	149 (3)
C7—H7*B*⋯O3^i^	0.97	2.49	3.167 (3)	127
C13—H13⋯O1^ii^	0.93	2.58	3.448 (3)	156
C21—H21⋯O6	0.93	2.42	3.206 (3)	143
C12—H12⋯*Cg*1^ii^	0.93	2.91	3.673 (2)	140
C17—H17⋯*Cg*1^i^	0.93	2.85	3.630 (3)	142

Symmetry codes: (i) 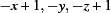; (ii) 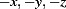.

**Fig. 1.**
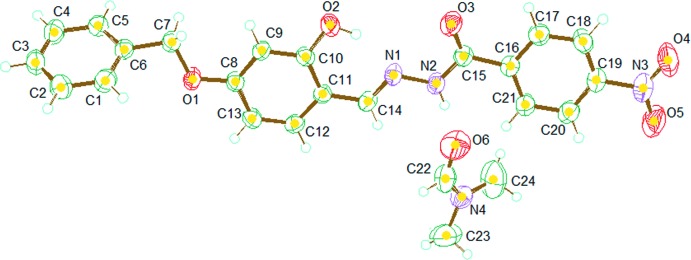
ORTEP view of the title compound drawn with 50% probability displacement ellipsoids for the non-H atoms.

**Fig. 2.**
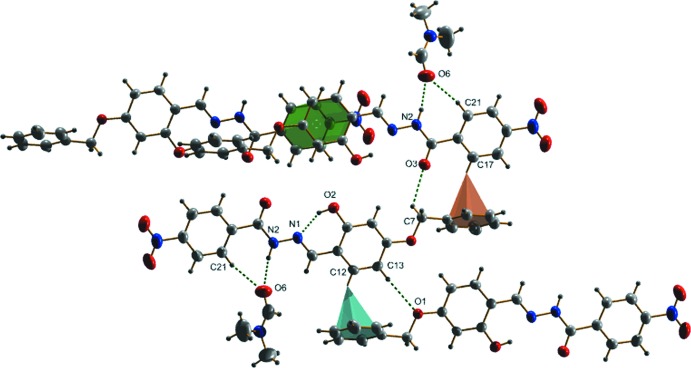
Hydrogen-bonding, C—H···π and π–π interactions present in the crystal structure of C21H17N3O5.C3H7NO.

**Fig. 3.**
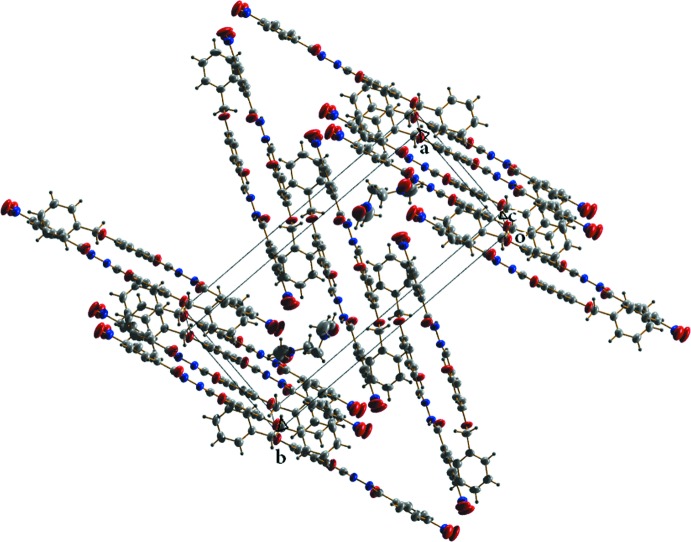
Packing diagram of the compound along c axis.

## supplementary crystallographic information

### Comment

Coordination chemistry and biochemistry of hydrazones have received increasing interest due to their chelating ability and their antimicrobial, antituberculosis and antitumour activities (Vicini *et al.* 2002; Savini *et al.*, 2002; Grande *et al.* 2007). As a continuous work on the hydrazide compounds, a new hydrazide compound, *N*'-[(*E*)-4-Benzyloxy-2-hydroxybenzylidene]-4-nitrobenzohydrazide dimethylformamide monosolvate, was prepared and structurally characterized.

The compound crystallizes in monoclinic space group *P*2_1_/c. This molecule adopts an *E* configuration (Fig.1) with respect to C14—N1 bond with torsional angles of 177.93 (18)°. The title compound exists in *amido* form with C15—O3 bond length of 1.217 (3) Å, which is very close to C═O bond length of a similar reported nitrobenzohydrazide compound (Joseph *et al.*, 2012). The aromatic ring (C1–C6) of the compound forms dihedral angles between the other two aromatic rings (C8–C13 and C16–C21) with angles of 67.63 (12) and 61.58 (12)° respectively. There are one classical N2—H2'···O6 and three non-classical C—H···O intermolecular hydrogen bonds (Fig. 2) present in the molecular system with D···A distances of 2.810 (3), 3.167 (3), 3.448 (3) and 3.206 (3) Å (Table 1) respectively. These intermolecular hydrogen bonds chain the molecules along *c* axis. Moreover, two C—H···π interactions between the H atoms attached at the C12 and C17 atoms and the corresponding aromatic ring C1–C6 of the neighbouring molecules with H···π distances of 3.673 (2) and 3.630 (3) Å respectively, also support the hydrogen bonding to form a one-dimensional layer along *c* axis. This supramolecular network is augmented by a weak π–π interaction (Fig. 2) between the phenyl rings (C8–C13 and C16–C21) of the molecules with a centroid–centroid distance of 3.650 (13) Å by interconnecting the molecules along *b* axis. Packing of molecules is predominantly favored by the classical intermolecular hydrogen bonding and C—H···π interactions. Other short ring interactions are very weak as they correspond to their centroid-centroid distances greater than 4 Å. Intramolecular classical hydrogen bond is also observed in the molecular system (Table 1). Fig. 3 shows the packing diagram of the title compound along *c* axis.

### Experimental

The title compound was prepared by adapting a reported procedure (Mathew & Kurup, 2011; Despaigne *et al.*, 2009). A methanolic solution of 4-nitrobenzohydrazide (0.181 g, 1 mmol) was added to a solution of 4-(benzyloxy)-2-hydroxybenzldehyde (0.228 g, 1 mmol) in ethanol and the reaction mixture was refluxed for 5 h after adding a few drops of dilute sulfuric acid. On cooling yellow colored crystals were collected, washed with few drops of methanol, and dried over P_4_O_10_*in vacuo*. Single crystals of the title compound suitable for X-ray analysis were obtained by recrystallization from a mixture of ethanol and dimethylformamide (1:1 *v*/*v*).

### Refinement

All H atoms on C were placed in calculated positions, guided by difference maps, with C–H bond distances 0.93–0.97 Å. H atoms were assigned as *U*_iso_=1.2Ueq (1.5 for Me). N2–H2' and O2–H2O H atoms were located from difference maps and restrained using *DFIX* instructions. Omitted owing to bad disagreement was the reflection (2 6 2).

### Crystal data

**Table:**

C_21_H_17_N_3_O_5_·C_3_H_7_NO	*F*(000) = 976
*M**_r_* = 464.47	*D*_x_ = 1.351 Mg m^−^^3^
Monoclinic, *P*2_1_/*c*	Mo *K*α radiation, λ = 0.71073 Å
Hall symbol: -P 2ybc	Cell parameters from 3347 reflections
*a* = 10.0160 (8) Å	θ = 2.7–27.6°
*b* = 22.661 (2) Å	µ = 0.10 mm^−^^1^
*c* = 10.2611 (11) Å	*T* = 296 K
β = 101.392 (5)°	Block, yellow
*V* = 2283.1 (4) Å^3^	0.40 × 0.20 × 0.20 mm
*Z* = 4	

### Data collection

**Table:**

Bruker Kappa APEXII CCD diffractometer	4910 independent reflections
Radiation source: fine-focus sealed tube	2901 reflections with *I* > 2σ(*I*)
Graphite monochromator	*R*_int_ = 0.031
ω and φ scan	θ_max_ = 27.0°, θ_min_ = 1.8°
Absorption correction: multi-scan (*SADABS*; Bruker, 2004)	*h* = −12→11
*T*_min_ = 0.977, *T*_max_ = 0.981	*k* = −28→28
16107 measured reflections	*l* = −13→8

### Refinement

**Table:**

Refinement on *F*^2^	Secondary atom site location: difference Fourier map
Least-squares matrix: full	Hydrogen site location: inferred from neighbouring sites
*R*[*F*^2^ > 2σ(*F*^2^)] = 0.053	H atoms treated by a mixture of independent and constrained refinement
*wR*(*F*^2^) = 0.176	*w* = 1/[σ^2^(*F*_o_^2^) + (0.0894*P*)^2^ + 0.2279*P*] where *P* = (*F*_o_^2^ + 2*F*_c_^2^)/3
*S* = 1.04	(Δ/σ)_max_ < 0.001
4910 reflections	Δρ_max_ = 0.40 e Å^−^^3^
318 parameters	Δρ_min_ = −0.22 e Å^−^^3^
2 restraints	Extinction correction: *SHELXL97* (Sheldrick, 2008), Fc^*^=kFc[1+0.001xFc^2^λ^3^/sin(2θ)]^-1/4^
Primary atom site location: structure-invariant direct methods	Extinction coefficient: 0.0078 (15)

### Special details

**Table:**

Geometry. All e.s.d.'s (except the e.s.d. in the dihedral angle between two l.s. planes) are estimated using the full covariance matrix. The cell e.s.d.'s are taken into account individually in the estimation of e.s.d.'s in distances, angles and torsion angles; correlations between e.s.d.'s in cell parameters are only used when they are defined by crystal symmetry. An approximate (isotropic) treatment of cell e.s.d.'s is used for estimating e.s.d.'s involving l.s. planes.
Refinement. Refinement of *F*^2^ against ALL reflections. The weighted *R*-factor *wR* and goodness of fit *S* are based on *F*^2^, conventional *R*-factors *R* are based on *F*, with *F* set to zero for negative *F*^2^. The threshold expression of *F*^2^ > σ(*F*^2^) is used only for calculating *R*-factors(gt) *etc*. and is not relevant to the choice of reflections for refinement. *R*-factors based on *F*^2^ are statistically about twice as large as those based on *F*, and *R*- factors based on ALL data will be even larger.

### Fractional atomic coordinates and isotropic or equivalent isotropic displacement parameters (Å^2^)

**Table:**

	*x*	*y*	*z*	*U*_iso_*/*U*_eq_	
O1	0.07170 (14)	−0.02077 (7)	0.19458 (16)	0.0520 (4)	
O2	0.50913 (16)	0.05541 (8)	0.38186 (17)	0.0554 (5)	
O3	0.87382 (16)	0.10480 (8)	0.40720 (18)	0.0605 (5)	
O4	1.4753 (2)	0.21761 (12)	0.2984 (3)	0.1028 (9)	
O5	1.4051 (2)	0.21112 (11)	0.0893 (3)	0.0926 (8)	
O6	0.7557 (2)	0.15662 (12)	−0.0661 (2)	0.0952 (8)	
N1	0.65997 (16)	0.08803 (8)	0.2177 (2)	0.0430 (5)	
N2	0.77984 (17)	0.10975 (8)	0.1910 (2)	0.0435 (5)	
N3	1.3896 (2)	0.20500 (9)	0.2026 (3)	0.0619 (6)	
N4	0.71736 (19)	0.18185 (9)	−0.2805 (2)	0.0540 (5)	
C1	−0.0899 (2)	−0.12417 (10)	0.2521 (3)	0.0543 (6)	
H1	−0.0142	−0.1398	0.2244	0.065*	
C2	−0.2068 (3)	−0.15693 (11)	0.2395 (3)	0.0631 (7)	
H2A	−0.2098	−0.1947	0.2036	0.076*	
C3	−0.3193 (2)	−0.13453 (11)	0.2792 (3)	0.0574 (7)	
H3	−0.3983	−0.1570	0.2701	0.069*	
C4	−0.3152 (2)	−0.07925 (11)	0.3320 (3)	0.0544 (6)	
H4	−0.3911	−0.0639	0.3599	0.065*	
C5	−0.1976 (2)	−0.04604 (10)	0.3440 (2)	0.0453 (6)	
H5	−0.1955	−0.0081	0.3787	0.054*	
C6	−0.0841 (2)	−0.06820 (9)	0.3056 (2)	0.0407 (5)	
C7	0.0453 (2)	−0.03359 (10)	0.3232 (2)	0.0459 (6)	
H7A	0.0366	0.0028	0.3707	0.055*	
H7B	0.1199	−0.0563	0.3742	0.055*	
C8	0.19366 (19)	0.00386 (9)	0.1859 (2)	0.0389 (5)	
C9	0.2933 (2)	0.01794 (9)	0.2933 (2)	0.0407 (5)	
H9	0.2796	0.0112	0.3791	0.049*	
C10	0.41497 (19)	0.04237 (9)	0.2735 (2)	0.0378 (5)	
C11	0.43622 (19)	0.05309 (9)	0.1444 (2)	0.0370 (5)	
C12	0.3322 (2)	0.03864 (10)	0.0385 (2)	0.0438 (5)	
H12	0.3443	0.0459	−0.0477	0.053*	
C13	0.2125 (2)	0.01410 (10)	0.0570 (2)	0.0443 (5)	
H13	0.1446	0.0044	−0.0156	0.053*	
C14	0.5624 (2)	0.07781 (9)	0.1208 (2)	0.0413 (5)	
H14	0.5722	0.0862	0.0344	0.050*	
C15	0.8836 (2)	0.11689 (9)	0.2939 (2)	0.0409 (5)	
C16	1.0138 (2)	0.14008 (9)	0.2640 (2)	0.0389 (5)	
C17	1.1128 (2)	0.15662 (10)	0.3697 (3)	0.0504 (6)	
H17	1.0960	0.1535	0.4554	0.060*	
C18	1.2367 (2)	0.17777 (11)	0.3517 (3)	0.0536 (6)	
H18	1.3035	0.1890	0.4239	0.064*	
C19	1.2585 (2)	0.18182 (9)	0.2247 (3)	0.0456 (6)	
C20	1.1643 (2)	0.16467 (12)	0.1179 (3)	0.0596 (7)	
H20	1.1825	0.1672	0.0326	0.072*	
C21	1.0410 (2)	0.14332 (12)	0.1380 (3)	0.0566 (7)	
H21	0.9756	0.1310	0.0656	0.068*	
C22	0.7086 (3)	0.14686 (13)	−0.1811 (4)	0.0675 (8)	
H22	0.6620	0.1115	−0.2011	0.081*	
C23	0.6524 (4)	0.1653 (2)	−0.4133 (4)	0.1072 (13)	
H23A	0.6184	0.1257	−0.4128	0.161*	
H23B	0.7172	0.1674	−0.4706	0.161*	
H23C	0.5782	0.1917	−0.4450	0.161*	
C24	0.7845 (4)	0.23712 (15)	−0.2607 (5)	0.1139 (15)	
H24A	0.8578	0.2379	−0.3085	0.171*	
H24B	0.8202	0.2428	−0.1676	0.171*	
H24C	0.7210	0.2681	−0.2927	0.171*	
H2'	0.784 (2)	0.1204 (10)	0.1096 (12)	0.046 (7)*	
H2O	0.580 (2)	0.0665 (14)	0.355 (3)	0.101 (12)*	

### Atomic displacement parameters (Å^2^)

**Table:**

	*U*^11^	*U*^22^	*U*^33^	*U*^12^	*U*^13^	*U*^23^
O1	0.0393 (8)	0.0784 (11)	0.0368 (10)	−0.0205 (7)	0.0040 (7)	0.0106 (8)
O2	0.0456 (9)	0.0794 (12)	0.0386 (10)	−0.0217 (8)	0.0021 (8)	0.0011 (9)
O3	0.0497 (9)	0.0889 (13)	0.0435 (11)	−0.0115 (8)	0.0101 (8)	0.0135 (10)
O4	0.0555 (12)	0.156 (2)	0.093 (2)	−0.0442 (13)	0.0046 (12)	0.0068 (17)
O5	0.0732 (14)	0.1240 (19)	0.0883 (19)	−0.0293 (12)	0.0346 (13)	0.0187 (15)
O6	0.0904 (16)	0.135 (2)	0.0553 (15)	0.0031 (13)	0.0032 (13)	0.0299 (15)
N1	0.0351 (9)	0.0479 (10)	0.0477 (13)	−0.0049 (7)	0.0121 (9)	0.0053 (9)
N2	0.0372 (9)	0.0523 (11)	0.0428 (13)	−0.0069 (8)	0.0121 (9)	0.0068 (10)
N3	0.0455 (12)	0.0616 (13)	0.0805 (19)	−0.0063 (9)	0.0169 (13)	0.0116 (13)
N4	0.0539 (11)	0.0587 (12)	0.0504 (14)	0.0043 (9)	0.0130 (10)	0.0082 (11)
C1	0.0560 (14)	0.0531 (14)	0.0583 (18)	−0.0020 (11)	0.0222 (13)	−0.0001 (12)
C2	0.0785 (18)	0.0465 (13)	0.069 (2)	−0.0189 (12)	0.0264 (16)	−0.0068 (13)
C3	0.0500 (13)	0.0650 (16)	0.0575 (18)	−0.0217 (11)	0.0111 (12)	0.0049 (14)
C4	0.0387 (12)	0.0662 (16)	0.0592 (18)	−0.0036 (10)	0.0115 (12)	0.0052 (13)
C5	0.0458 (12)	0.0472 (12)	0.0432 (15)	−0.0044 (9)	0.0094 (10)	0.0019 (11)
C6	0.0391 (11)	0.0490 (12)	0.0335 (13)	−0.0055 (9)	0.0056 (9)	0.0066 (10)
C7	0.0397 (11)	0.0599 (14)	0.0375 (14)	−0.0100 (9)	0.0064 (10)	0.0058 (11)
C8	0.0350 (10)	0.0448 (12)	0.0361 (13)	−0.0052 (8)	0.0049 (9)	0.0061 (10)
C9	0.0410 (11)	0.0499 (12)	0.0323 (13)	−0.0087 (9)	0.0096 (10)	0.0051 (10)
C10	0.0358 (10)	0.0409 (11)	0.0351 (13)	−0.0031 (8)	0.0035 (9)	0.0005 (10)
C11	0.0344 (10)	0.0412 (11)	0.0359 (13)	0.0003 (8)	0.0082 (9)	0.0017 (9)
C12	0.0442 (12)	0.0561 (13)	0.0319 (13)	−0.0037 (9)	0.0095 (10)	0.0047 (10)
C13	0.0376 (11)	0.0578 (13)	0.0345 (13)	−0.0071 (9)	−0.0001 (10)	0.0023 (11)
C14	0.0398 (11)	0.0458 (12)	0.0407 (14)	−0.0005 (9)	0.0135 (10)	0.0046 (10)
C15	0.0383 (11)	0.0431 (12)	0.0418 (15)	−0.0002 (8)	0.0087 (10)	0.0048 (10)
C16	0.0374 (10)	0.0377 (11)	0.0421 (14)	0.0013 (8)	0.0091 (10)	0.0038 (10)
C17	0.0484 (13)	0.0608 (14)	0.0415 (15)	−0.0074 (10)	0.0078 (11)	0.0054 (12)
C18	0.0439 (13)	0.0612 (15)	0.0518 (17)	−0.0110 (10)	−0.0003 (12)	0.0038 (12)
C19	0.0371 (11)	0.0424 (12)	0.0585 (17)	−0.0011 (9)	0.0125 (11)	0.0057 (11)
C20	0.0502 (14)	0.0867 (18)	0.0447 (17)	−0.0078 (12)	0.0162 (13)	0.0039 (14)
C21	0.0411 (12)	0.0855 (18)	0.0430 (16)	−0.0138 (11)	0.0081 (11)	−0.0016 (13)
C22	0.0493 (15)	0.0691 (17)	0.083 (3)	0.0012 (12)	0.0110 (16)	0.0108 (18)
C23	0.091 (2)	0.169 (4)	0.060 (2)	0.023 (2)	0.0116 (19)	−0.025 (2)
C24	0.099 (3)	0.071 (2)	0.177 (5)	−0.0164 (18)	0.041 (3)	0.016 (2)

### Geometric parameters (Å, º)

**Table:**

O1—C8	1.362 (2)	C8—C9	1.371 (3)
O1—C7	1.426 (3)	C8—C13	1.392 (3)
O2—C10	1.341 (3)	C9—C10	1.389 (3)
O2—H2O	0.847 (10)	C9—H9	0.9300
O3—C15	1.217 (3)	C10—C11	1.403 (3)
O4—N3	1.205 (3)	C11—C12	1.388 (3)
O5—N3	1.211 (3)	C11—C14	1.446 (3)
O6—C22	1.201 (4)	C12—C13	1.369 (3)
N1—C14	1.271 (3)	C12—H12	0.9300
N1—N2	1.374 (2)	C13—H13	0.9300
N2—C15	1.337 (3)	C14—H14	0.9300
N2—H2'	0.879 (10)	C15—C16	1.493 (3)
N3—C19	1.472 (3)	C16—C17	1.370 (3)
N4—C22	1.308 (4)	C16—C21	1.375 (3)
N4—C24	1.417 (4)	C17—C18	1.376 (3)
N4—C23	1.439 (4)	C17—H17	0.9300
C1—C2	1.371 (3)	C18—C19	1.367 (3)
C1—C6	1.379 (3)	C18—H18	0.9300
C1—H1	0.9300	C19—C20	1.354 (4)
C2—C3	1.369 (4)	C20—C21	1.380 (3)
C2—H2A	0.9300	C20—H20	0.9300
C3—C4	1.363 (3)	C21—H21	0.9300
C3—H3	0.9300	C22—H22	0.9300
C4—C5	1.383 (3)	C23—H23A	0.9600
C4—H4	0.9300	C23—H23B	0.9600
C5—C6	1.370 (3)	C23—H23C	0.9600
C5—H5	0.9300	C24—H24A	0.9600
C6—C7	1.495 (3)	C24—H24B	0.9600
C7—H7A	0.9700	C24—H24C	0.9600
C7—H7B	0.9700		
			
C8—O1—C7	118.47 (17)	C10—C11—C14	121.7 (2)
C10—O2—H2O	107 (2)	C13—C12—C11	122.0 (2)
C14—N1—N2	118.4 (2)	C13—C12—H12	119.0
C15—N2—N1	117.33 (19)	C11—C12—H12	119.0
C15—N2—H2'	122.7 (14)	C12—C13—C8	119.1 (2)
N1—N2—H2'	119.9 (14)	C12—C13—H13	120.5
O4—N3—O5	123.4 (2)	C8—C13—H13	120.5
O4—N3—C19	118.2 (2)	N1—C14—C11	120.1 (2)
O5—N3—C19	118.4 (2)	N1—C14—H14	119.9
C22—N4—C24	121.8 (3)	C11—C14—H14	119.9
C22—N4—C23	119.5 (3)	O3—C15—N2	122.03 (19)
C24—N4—C23	118.6 (3)	O3—C15—C16	120.9 (2)
C2—C1—C6	120.3 (2)	N2—C15—C16	117.0 (2)
C2—C1—H1	119.9	C17—C16—C21	118.9 (2)
C6—C1—H1	119.9	C17—C16—C15	117.3 (2)
C3—C2—C1	120.6 (2)	C21—C16—C15	123.7 (2)
C3—C2—H2A	119.7	C16—C17—C18	121.3 (2)
C1—C2—H2A	119.7	C16—C17—H17	119.3
C4—C3—C2	119.8 (2)	C18—C17—H17	119.3
C4—C3—H3	120.1	C19—C18—C17	118.0 (2)
C2—C3—H3	120.1	C19—C18—H18	121.0
C3—C4—C5	119.6 (2)	C17—C18—H18	121.0
C3—C4—H4	120.2	C20—C19—C18	122.4 (2)
C5—C4—H4	120.2	C20—C19—N3	118.5 (2)
C6—C5—C4	121.0 (2)	C18—C19—N3	119.1 (2)
C6—C5—H5	119.5	C19—C20—C21	118.7 (2)
C4—C5—H5	119.5	C19—C20—H20	120.6
C5—C6—C1	118.66 (19)	C21—C20—H20	120.6
C5—C6—C7	121.5 (2)	C16—C21—C20	120.6 (2)
C1—C6—C7	119.9 (2)	C16—C21—H21	119.7
O1—C7—C6	108.03 (18)	C20—C21—H21	119.7
O1—C7—H7A	110.1	O6—C22—N4	125.5 (3)
C6—C7—H7A	110.1	O6—C22—H22	117.3
O1—C7—H7B	110.1	N4—C22—H22	117.3
C6—C7—H7B	110.1	N4—C23—H23A	109.5
H7A—C7—H7B	108.4	N4—C23—H23B	109.5
O1—C8—C9	124.2 (2)	H23A—C23—H23B	109.5
O1—C8—C13	114.98 (19)	N4—C23—H23C	109.5
C9—C8—C13	120.79 (19)	H23A—C23—H23C	109.5
C8—C9—C10	119.7 (2)	H23B—C23—H23C	109.5
C8—C9—H9	120.2	N4—C24—H24A	109.5
C10—C9—H9	120.2	N4—C24—H24B	109.5
O2—C10—C9	117.4 (2)	H24A—C24—H24B	109.5
O2—C10—C11	122.09 (18)	N4—C24—H24C	109.5
C9—C10—C11	120.6 (2)	H24A—C24—H24C	109.5
C12—C11—C10	117.91 (18)	H24B—C24—H24C	109.5
C12—C11—C14	120.3 (2)		
			
C14—N1—N2—C15	−176.17 (19)	C9—C8—C13—C12	−0.1 (3)
C6—C1—C2—C3	0.3 (4)	N2—N1—C14—C11	177.93 (18)
C1—C2—C3—C4	−0.2 (4)	C12—C11—C14—N1	−176.3 (2)
C2—C3—C4—C5	0.6 (4)	C10—C11—C14—N1	3.1 (3)
C3—C4—C5—C6	−1.1 (4)	N1—N2—C15—O3	0.0 (3)
C4—C5—C6—C1	1.2 (4)	N1—N2—C15—C16	179.57 (17)
C4—C5—C6—C7	−177.5 (2)	O3—C15—C16—C17	−10.9 (3)
C2—C1—C6—C5	−0.8 (4)	N2—C15—C16—C17	169.57 (19)
C2—C1—C6—C7	177.9 (2)	O3—C15—C16—C21	166.3 (2)
C8—O1—C7—C6	−171.59 (18)	N2—C15—C16—C21	−13.2 (3)
C5—C6—C7—O1	−115.3 (2)	C21—C16—C17—C18	1.8 (4)
C1—C6—C7—O1	66.1 (3)	C15—C16—C17—C18	179.2 (2)
C7—O1—C8—C9	−0.8 (3)	C16—C17—C18—C19	−0.1 (4)
C7—O1—C8—C13	178.96 (19)	C17—C18—C19—C20	−1.4 (4)
O1—C8—C9—C10	179.29 (19)	C17—C18—C19—N3	179.2 (2)
C13—C8—C9—C10	−0.4 (3)	O4—N3—C19—C20	−176.6 (3)
C8—C9—C10—O2	179.93 (19)	O5—N3—C19—C20	4.3 (3)
C8—C9—C10—C11	0.4 (3)	O4—N3—C19—C18	2.7 (3)
O2—C10—C11—C12	−179.36 (19)	O5—N3—C19—C18	−176.4 (2)
C9—C10—C11—C12	0.1 (3)	C18—C19—C20—C21	1.2 (4)
O2—C10—C11—C14	1.3 (3)	N3—C19—C20—C21	−179.5 (2)
C9—C10—C11—C14	−179.25 (19)	C17—C16—C21—C20	−2.1 (4)
C10—C11—C12—C13	−0.7 (3)	C15—C16—C21—C20	−179.3 (2)
C14—C11—C12—C13	178.7 (2)	C19—C20—C21—C16	0.6 (4)
C11—C12—C13—C8	0.7 (3)	C24—N4—C22—O6	−0.9 (4)
O1—C8—C13—C12	−179.87 (19)	C23—N4—C22—O6	−178.2 (3)

### Hydrogen-bond geometry (Å, º)

Cg1 is the centroid of the C1-C6 ring.

**Table:**

*D*—H···*A*	*D*—H	H···*A*	*D*···*A*	*D*—H···*A*
N2—H2′···O6	0.88 (1)	1.95 (1)	2.810 (3)	166 (2)
O2—H2*O*···N1	0.85 (1)	1.82 (2)	2.583 (2)	149 (3)
C7—H7*B*···O3^i^	0.97	2.49	3.167 (3)	127
C13—H13···O1^ii^	0.93	2.58	3.448 (3)	156
C21—H21···O6	0.93	2.42	3.206 (3)	143
C12—H12···*Cg*1^ii^	0.93	2.91	3.673 (2)	140
C17—H17···*Cg*1^i^	0.93	2.85	3.630 (3)	142

Symmetry codes: (i) −*x*+1, −*y*, −*z*+1; (ii) −*x*, −*y*, −*z*.
